# Cyberknife^®^ hypofractionated stereotactic radiosurgery (CK-hSRS) as salvage treatment for brain metastases

**DOI:** 10.1007/s00432-021-03564-z

**Published:** 2021-02-26

**Authors:** Sergej Telentschak, Daniel Ruess, Stefan Grau, Roland Goldbrunner, Niklas von Spreckelsen, Karolina Jablonska, Harald Treuer, Martin Kocher, Maximilian Ruge

**Affiliations:** 1 Department of Stereotaxy and Functional Neurosurgery, Centre of Neurosurgery, Faculty of Medicine and University Hospital, University of Cologne, Cologne, 50937 Germany; 2 Department of General Neurosurgery, Centre of Neurosurgery, Faculty of Medicine and University Hospital, University of Cologne, Cologne, 50937 Germany; 3 Department of Radiation Oncology, Faculty of Medicine and University Hospital, University of Cologne, Cologne, 50937 Germany

**Keywords:** Cyberknife, Hypofractionated stereotactical radiosurgery, Brain metastasis, Salvage treatment, Neurooncology

## Abstract

**Purpose:**

The introduction of hypofractionated stereotactic radiosurgery (hSRS) extended the treatment modalities beyond the well-established single-fraction stereotactic radiosurgery and fractionated radiotherapy. Here, we report the efficacy and side effects of hSRS using Cyberknife^®^ (CK-hSRS) for the treatment of patients with critical brain metastases (BM) and a very poor prognosis. We discuss our experience in light of current literature.

**Methods:**

All patients who underwent CK-hSRS over 3 years were retrospectively included. We applied a surface dose of 27 Gy in 3 fractions. Rates of local control (LC), systemic progression-free survival (PFS), and overall survival (OS) were estimated using Kaplan–Meier method. Treatment-related complications were rated using the Common Terminology Criteria for Adverse Events (CTCAE).

**Results:**

We analyzed 34 patients with 75 BM. 53% of the patients had a large tumor, tumor location was eloquent in 32%, and deep seated in 15%. 36% of tumors were recurrent after previous irradiation. The median Karnofsky Performance Status was 65%.

The actuarial rates of LC at 3, 6, and 12 months were 98%, 98%, and 78.6%, respectively.

Three, 6, and 12 months PFS was 38%, 32%, and 15%, and OS was 65%, 47%, and 28%, respectively. Median OS was significantly associated with higher KPS, which was the only significant factor for survival. Complications CTCAE grade 1–3 were observed in 12%.

**Conclusion:**

Our radiation schedule showed a reasonable treatment effectiveness and tolerance. Representing an optimal salvage treatment for critical BM in patients with a very poor prognosis and clinical performance state, CK-hSRS may close the gap between surgery, stereotactic radiosurgery, conventional radiotherapy, and palliative care.

## Introduction

Single fraction stereotactic radiosurgery (SRS) is a well-established part of standard care in BM. Its application is limited by tumor size (cross-sectional diameter > 3 cm), the number of targets, and a close proximity to critical structures (i.e., brainstem, cerebellar nuclei; sensorimotor, language or visual cortex; basal ganglia, hypothalamus or thalamus; internal capsule; optic pathway). A pre-irradiation [e.g., SRS, brachytherapy, fractionated radiation therapy (fRT), or WBRT] can limit the possible radiation dose. Cyberknife^®^ hSRS may overcome these limitations by combining the advantages of SRS (i.e., shorter treatment period, steep dose decay) with the radiobiological advantages of fRT in terms of re-oxygenation of hypoxic tumor tissue, redistribution of the cell cycle to a more sensitive phase, and sparing of radio-sensible adjacent brain structures by lower doses (Brenner et al. 1991). Thus, hypofractionated stereotactic radiosurgery (hSRS) extends the indications of stereotactic radiation techniques and of fractionated radiotherapy beyond the previous constraints.

To date, clinical data regarding feasibility, local control, and toxicity of Cyberknife® hSRS (CK-hSRS) for the treatment of BM are scarce.

## Materials and methods

### Subjects and patients

In this single center retrospective analysis, we included all patients who underwent CK-hSRS between September 2014 and August 2017. We queried our database for demographic, disease, and treatment-related parameters. Recursive partitioning analysis (RPA) classification was determined for all patients according to Gaspar et al. (1997).

### Indications for CK-hSRS

Decision for hSRS was made by an interdisciplinary neurooncological tumor board. The treatment indications were: (1) BM located in close proximity to or in eloquent brain areas, (2) deep-seated BM in recurrence after previous irradiation, and (3) large BM (> 3 cm) if surgery is not feasible. Additional small BMs were co-treated in the same setting to simplify the treatment management. Neurological or radiological evidence of carcinomatous meningitis was an exclusion criterion. Patients with a poor Karnofsky performance status (< 40%) or without further oncological options were not treated with this protocol. Informed consent was obtained from all patients.

### Follow-up and end points

Clinical assessment and follow-up MRI were carried out in at least 3 month intervals or earlier in cases of significant neurologic deterioration. The tumor size was defined as the largest cross-sectional diameter on T1-weighted contrast-enhanced axial sequences. Each lesion was measured for local tumor response and graded using the RANO criteria for brain metastases: local tumor control (LC) was defined as either complete remission, partial remission or stable disease. An increase in tumor size ≥ 30% was suspected as local failure a progressive disease (Lin et al. 2015). To rule out a pseudo-progress a short-term follow-up MRI, [18F]-fluoro-ethyl-l-tyrosine PET (FET-PET) or stereotactical biopsy was performed in confounding cases. We evaluated systemic progression-free survival (PFS), overall survival (OS), and treatment related early and late adverse events according to Common Terminology Criteria for Adverse Events (CTCAE) version 4.03. Cause of the death was classified due to primary disease and comorbidities, neurological disease, or unknown. Neurological death was defined as death from any impact of intracranial metastases, e.g., tumor recurrence, carcinomatous meningitis, or cerebral dissemination.

### Radiosurgery technique

A high-resolution contrast-enhanced cranial computed tomography (CCT) and MRI scans were obtained a few days before SRS. We used an MRI protocol consisting of 4 MRI modalities: T1-weighted contrast-enhanced axial sequences with 2 mm sl (3D T1) and coronal sequences with 1.2 mm sl (3D T1w FFE), T2-weighted axial sequences with 2 mm sl (T2 TSE), and axial FLAIR sequences with 2 mm sl (FLAIR long TR). The Cyberknife^®^ treatment planning was carried out with the software Multiplan v4.5 (MultiPlan, Inc., New York, USA). The tumor targets and the critical structures were contoured on stereotactic MRI scans (1.5 or 3 T; Philips, Hamburg, Germany), which were obtained a few days before hSRS. PTV was outlined according to a suspected CTV as GTV with a 1–2 mm margin. The cMRI scans were fused with the stereotactic CCT (1 mm slice thickness [sl], Toshiba). For Cyberknife® treatment, the patient`s head was immobilized with a custom-made aquaplast mask.

Following a prospective protocol, we applied a surface dose of 27 Gy in three fractions prescribed on the 65% isodose. The prescribed surface dose (Gy), isodose level, mean dose, minimum dose, maximum dose, tumor coverage, homogeneity index, conformity index, new conformity index, VOI 16 (volume of healthy brain tissue that is irradiated with a total dose of at least 16 Gy), collimator, and the number of radiation beams were recorded. All treatments were rendered by an experienced team of SRS physicians and medical physicists.

### Statistical analysis

Statistical analyses were performed with SPSS, version 23 (IBM Corp., Armonk, New York, USA). Local control (LC), systemic progression-free survival (PFS), and overall survival (OS) were analyzed using the Kaplan–Meier method. Prognostic factors were identified by the log-rank test. A *p* value < 0.05 was considered statistically significant.

## Results

### Patient characteristics and follow-up

34 patients with 75 brain metastases were included (Table 1). Large tumor size was the main indication in 53% of patients (*n* = 18). Eloquent location of BM indicated hSRS in 32% (*n* = 11) and recurrent, deep-seated BM after previous irradiation in 15% (*n* = 5) of cases. We co-treated all small or not eloquent located additional BM to simplify the treatment management, sparing medical resources and patients time, or due to previous irradiation. Since more than 50% of patients harbored 2–5 BMs requiring treatment, the majority of the treated tumors were additional. All hSRS treatments were completed within 5–7 days. A total of 24 patients (70.6%) with 57 treated BM (76%) were evaluated by imaging follow-up (FU). The median radiological FU was 8.0 months (range 2–28). In 10 patients (29.4%) with 18 treated tumors (24%), FU imaging was not available due to clinical worsening and consecutive death. The median clinical FU for all 34 patients was 6 months (range 1–28).

**Table 1:** Clinical and radiological patient characteristics

Characteristics	Values
Total no. of treated patients	34
No. of patients with	
1 Brain metastasis	16
2 Brain metastases	6
3 Brain metastases	5
4 Brain metastases	3
5 Brain metastases	4
Median age (range, years)	55.5 (35–84)
Gender (m: f)	13: 21
Median KPS (range, %)	65 (50–90)
Median radiological follow-up (range, months)	8.0 (2–28)
Neurological symptoms	
Headache	10
Palsy	7
Seizure	7
Dysphasia	6
Disequilibrium	5
Nausea	2
P-fossa complaints	2
Sensory disturbance	1
Visual field loss	1
None	6
RPA class (I:II:III), no. of patients	7:10:17
Histology of primary sites [*n* (%)]:	
Lung	14 (41%)
Breast	11 (32%)
Colon	2 (6%)
Pancreas	2 (6%)
Melanoma	1 (3%)
Other	4 (12%)
Pre-irradiation [*n* (%)]:	
Local recurrence after WBRT	5 (14.7%)
New lesion after WBRT	4 (11.8%)
Local recurrence after other modalities	3 (8.8%)
No previous irradiation treatment	23 (67.6%)
Inclusion criteria [*n* (%)]:	
Large BM (i.e., ≥ 3 cm)	18 (53%)
Eloquent located	11 (32%)
Deep seated, recurrent after irradiation	5 (15%)

### Tumor characteristics

Details of the treated tumors are summarized in Table 2. The median maximum tumor diameter was 2.0 cm (range 0.4–4.3 cm) and the median tumor volume in terms of PTV amounted to 4.6 cm^3^ (range 0.03–24.8 cm).

**Table 2:** Tumor characteristics

Characteristics	values
Total no. of treated tumors (%)	75 (100%)
No. of treated tumors with available imaging FU (%)	57 (76%)
Median maximum tumor diameter (range, cm)	2.0 (0.4–4.3)
Median PTV (range, cm^3^)	4.6 (0.03–24.8)
Histology of primary sites [*n* (%)]:	
Lung	34 (45.3%)
Breast	30 (40%)
Colon	2 (2.7%)
Pancreas	2 (2.7%)
Melanoma	3 (4%)
Other	4 (5.3%)
Tumor location [*n* (%)]:	
Parietal lobe	25 (33.3%)
Frontal lobe	18 (24%)
Cerebellum	13 (17.3%)
Temporal lobe	7 (9.3%)
Occipital lobe	6 (8%)
Basal ganglia	4 (5.3%)
Brain stem	2 (2.7%)
Pre-irradiation [*n* (%)]:	
Local recurrence after WBRT	11 (15%)
New lesion after WBRT	13 (17%)
Local recurrence after other modalities	3 (4%)
No previous irradiation treatment	48 (64%)
Treatment details [*n* (%)]:	
Large BM (i.e., ≥ 3 cm)	18 (24%)
Eloquent located	11 (15%)
Deep seated, recurrent after irradiation	7 (9%)
Additionally co-treated small BM	39 (52%)

In 48 BM (64%), CK-hSRS was carried out as de-novo treatment. 27 BM (36%) were recurrences after irradiation, including 13 new distant lesions (17%) after WBRT, 11 locally recurrent lesions (15%) after WBRT, and 3 locally recurrent lesions (4%) after other irradiation modalities (brachytherapy and single shot radiosurgery).

### Local tumor control (LC)

**Fig. 1:**
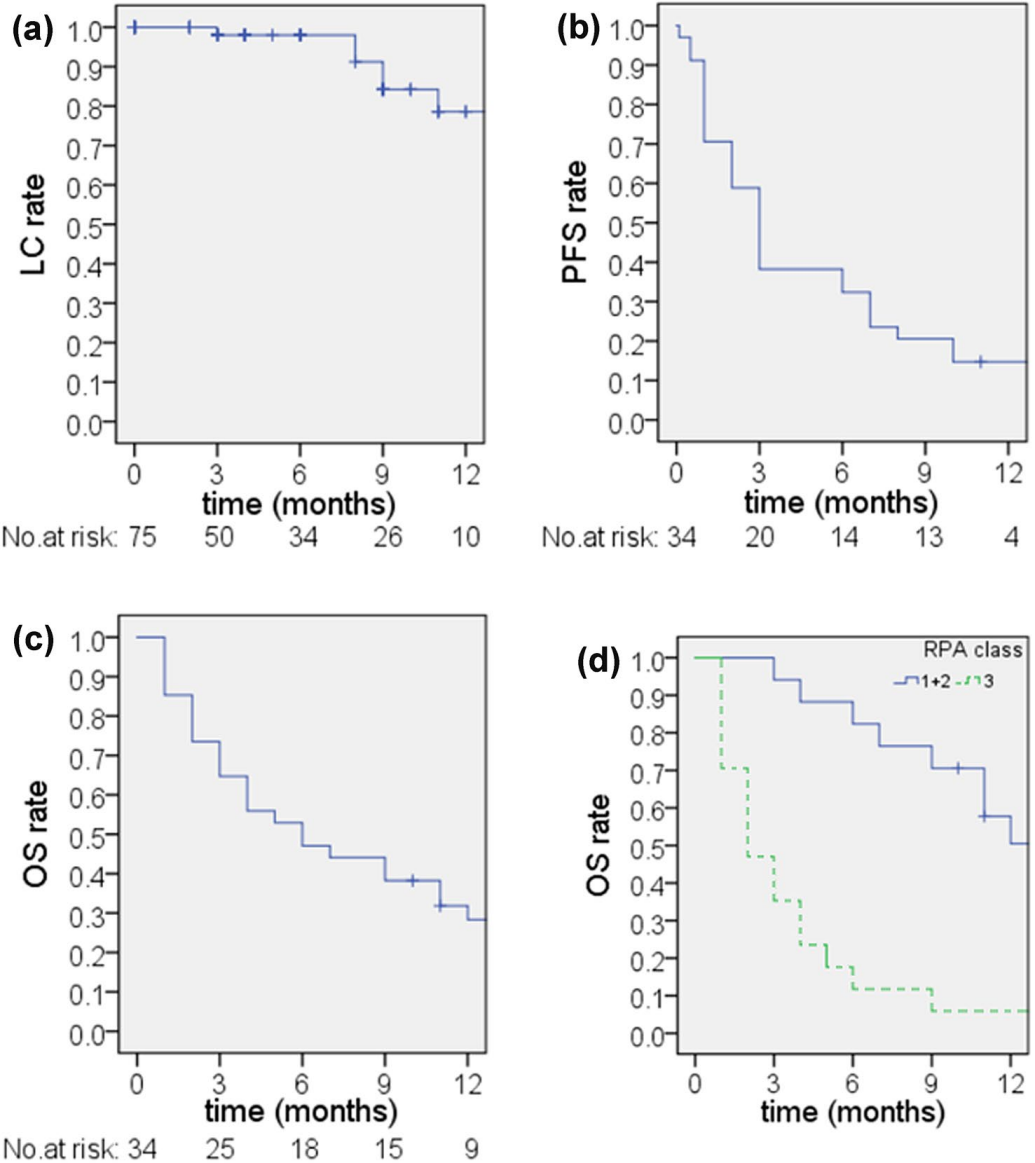
Kaplan–Meier estimates. **a** Actuarial local tumor control rates: at 3 months, at 6 months = 98%, and at 12 months = 78.6%. **b** Systemic progression-free survival rates: at 3 months = 38%, 6 months = 32%, and 12 months = 15%. **c** Overall survival rates: at 3 months = 65%, 6 months = 47%, and 12 months = 28%. **d** Overall Survival rates in RPA classes at 6 months after CK-hSRS: 82% in class 1 + 2 (median OS = 14 months, 95% CI 8.8–19.1 months) and 12% in class 3 (median OS = 2 months, 95% CI 0.7–3.3 months), *p* < 0.01

The actuarial radiologic local tumor control rate was 98% at 3 months as well as at 6 months, and 78.6% at 12 month (Fig. 1a).

Overall LC failure was observed in 6 of 57 (10.5%) tumors and in 5 of 24 patients (20.8%) with complete follow-up. Two of these patients were subsequently treated with surgical resection and adjuvant RT of the cavity. Another patient was treated with WBRT and a boost on recurrent metastases after the histological proof by stereotactical biopsy and the other two patients were treated in a purely palliative setting due to extracranially progressive disease. The median time interval between hSRS and the diagnosis of local control failure was 8 months (range 3–11).

According to RANO criteria for brain metastasis (Lin et al. 2015), partial remission was found in 33.3% of patients (*n* = 8/24) and stable disease in 12.5% (*n* = 6/24). Progressive disease was found in 54.2% (*n* = 13/24), with the majority (33.3%, *n* = 8/24) due to new brain metastases (i.e., distant brain control failure) and fewer due to local tumor control failure (20.8%, *n* = 5/24). In terms of the individual tumors, 5.3% (*n* = 3/57) were in complete remission, 68.4% (*n* = 39/57) were in a partial remission, 15.8% (*n* = 9/57) were stable, and 10.5% (*n* = 6/57) showed a local progression at last radiologic follow-up. Complete local remission was only observed in tumors less than 0.6 cm and local progressive disease occurred only in tumors exceeding at least 2 cm in the maximum diameter or a PTV of 4.6 cm^3^ (Table 3).

**Table 3:** Radiological appearance or local treatment status of particular hSRS-treated tumors at the last available radiological follow-up based on RANO criteria for brain metastasis (Lin et al. 2015) and the respective tumor diameters prior to CK-hSRS

Initial maximum tumor diameter prior to hSRS	Complete local remission (*n* = 3/57)	Partial local remission (*n* = 39/57)	Stable local state (*n* = 9/57)	Local progression (*n* = 6/57)
Median value (cm)	0.6	1.8	2.2	2.7
Mean value (cm)	0.5	2.1	2.2	2.8
± SD	0.1	1.2	0.9	0.5
Range (cm)	0.4–**0.6**	0.4–4.3	0.6–3.3	**2.0**–3.3

Important values are given in bold

### Systemic progression-free survival, overall survival, and death events

PFS rates were 38%, 32% and 15% at 3, 6, and 12 months, respectively (Fig. 1b). The median PFS amounted to 3 months. OS rates were 65%, 47%, and 28% at 3, 6, and 12 months, respectively (Fig. 1c). The median OS amounted to 6 months.

All patients were assessed in terms of RPA classification and were divided in class I (*n* = 7), class II (*n* = 10), and class III (*n* = 17) according to Gaspar et al. (1997). Since the patient number in class I was very low and the status of the extra-cranial disease was not always up to date, we merged class I with class II comparing them together with class III. There was a significant inter-class difference of overall survival rates based on Kaplan–Meier Curves (*p* < 0.01), the median overall survival was 14 months in RPA class I + II patients and 2 months in class III (Fig. 1d).

KPS was the only factor, which was significantly associated with survival. The BM count, PTV, prior WBRT, and age were not significant (Table 4).

**Table 4:** Factors related to OS, estimated by univariate log-rank analysis

Factors	Median OS (95% CI), months, respectively	Significance
Age < 65Y VS ≥ 65Y	6 (2.6–9.4) vs 9 (0–27)	*p* = 0.57
KPS ≥ 70% VS < 70%	14 (8.8–19.2) vs 2 (0.7–3.3)	***p < 0.01***
No prior WBRT VS prior WBRT	7 (2–12) vs 3 (0.2–5.8)	*p* = 0.39
Number of BM: 1 VS ≥ 2	6 (0–12.5) vs 5 (0.8–9.2)	*p* = 0.87
PTV < 14 ml VS > 14 ml	5 (0–10) vs 6 (0.6–11.4)	*p* = 0.19

Important values are given in bold

At last follow-up, 79% of patients had died (*n* = 27/34). The cause of death was extra-cranial progression of the primary or through other comorbidities in 65% (*n* = 22/34), distant meningeosis in 6% (*n* = 2/34), and unknown in 9% (*n* = 3/34).

### Adverse events and toxicities

Overall, four patients (12%) experienced early (< 6 weeks after hSRS) or late (> 6 weeks after hSRS) adverse events grade 1–3 according to CTCAE.

Among early complications (*n* = 2/34, 6%), one patient suffered a cerebral edema with a need for steroid treatment (CTCAE grade 2). Another patient had a deterioration of hemiparesis and additional structural focal epilepsy, which led to hospitalization and medical treatment (CTCAE grade 3).

Among late complications (*n* = 2/34, 6%), there was one asymptomatic patient with an extensive radiogenic edema, but no need for treatment (CTCAE grade 1) and another patient experienced severe headache and nausea as a cause of a radiation necrosis with an extensive edema and a need of hospitalization (CTCAE grade 3). All above-mentioned patients improved during medical therapy.

Ultimately, there was no case of permanent morbidity as a result of treatment.

## Discussion

Hypofractionated stereotactic radiosurgery by Cyberknife® (CK-hSRS) is often confused with hypofractionated stereotactic radiotherapy (HFSRT, by linear accelerator). Since 1993, clinical application of fractionated stereotactic radiotherapy for brain metastases performed by LINAC was extensively described in the literature (Salles et al. 1993; Manning et al. 2000; Feuvret et al. 2014; Lehrer et al. 2019; Rajakesari et al. 2014; Rades et al. 2017; Baliga et al. 2017; Aoyama et al. 2003; Ernst-Stecken et al. 2006; Aoki et al. 2006; Narayana et al. 2007; Kwon et al. 2009; Saitoh et al. 2010; Kim et al. 2011; Minniti et al. 2014). However, there are only few heterogeneous reports about the CK-hSRS for BM elucidating its application in different patient populations and with different study specifics (Table 5) (Inoue et al. 2014; Murai et al. 2014; Royer et al. 2017; Nakamura et al. 2017; Lesueur et al. 2018; Guan et al. 2020; Mengue et al. 2020). It is difficult to compare the results due to different study designs.

**Table 5:** Evidence on application of the CK-hSRS for treatment of patients with brain metastases

Authors (CK-hSRS)	CK-hSRS Sample size: patients/tumors	Study specifics	Tumor volume (cm^3^)	Maximum tumor diameter cm	Radiation schedule	Median KPS (range, %)	Median follow-up (range) months	median OS months	6 months LC %	12 months LC %	6 months OS %	12 months OS %	Adv. Events % (Grade, CTCAE)
Our data (Cologne, Germany)	34/75		0.03–24.8 (median 4.6)	0.4 – 4.3 (median 2)	27 Gy in 3 fr., IDL 65%	65 (50–90)	8.0 (2–28)	6	98	78.6	47	23	12 (G < 4)
Mengue et al. (2020) (France)	302/325	CK-hSRS alone vs. Surgery + CK-hSRS		0.6–6.5 (median 2.3)	3 × 9 Gy, 5 × 6 Gy or 5 × 7 Gy	~ 80			88.2	73.8		50.1	5
Guan et al. (2020) (China)	24/24;	+ Bevacizumab for recurrent pre-irradiated metastases	3.7–76.2 (median 17.3)		9.5–29 Gy In 2–5 fr IDL 62%-75%	60 (50–90)	13.5 (8–29)	18			100	87.5	37.5 (G < 4)
Lesueur et al. (2018) (France)	60/141 + 57;	CK-hSRS vs. SRS for melanoma & renal carcinoma	≤ 14.14 (median 0.7)	< 3	3 × 9–11 Gy 6 × 5–6 Gy IDL 80%	80 (50–100)	7.7	9.6	80	72	69	45	7.1 (G < 2)
Nakamura et al. (2017) (Japan)	20/26;	Only brainstem metastases		0.3–1.78 (median 0.8)	18–30 Gy in 3–5 fr IDL 70–80%	90% (50–100)	6.5 (0.5–38.0)	17	100	90	72	53	25 (G < 4)
Royer et al. 2017 (2017) (France)	76/124;	CK-hSRS only vs. SRS + WBRT (Article in French)		0.5–4.2 (median 1.85)	23.1 Gy in 3 fr IDL 70%	90% (60–100)	18.8 (1–69)	21.5					12% (G < 4)
Murai et al. (2014) (Japan)	54/61		≥ 8 to < 33; ≥ 33	2.5—< 4; ≥ 4	18–30 Gy in 3 fract.; 21–35 Gy in 5 fract	≥ 70% *n* = 41, < 70% *n* = 13		6	77	69	52	31	7 (G < 3)
Inoue et al. (2014) (Japan)	88/92		10–74.6–(median 16.2)		25-40 Gy in 3–10 fr IDL 57–55%	70% (50–100)	7 (3–41)	9			74	43	11 (G < 3)

In accordance to previous reports, tumor size was the main indication for treatment in our collective on the basis of poor performance state or patient’s will, while eloquent location and deep-seated BM recurrences were indications, due to limits of conventional treatment modalities.

The median FU of our patients and the actuarial LC rates of the treated tumors are in the range of the current literature on the CK-hSRS (Table 5). Longer reported FU may result from a better performance state of treated patients.

In comparison to other series, our OS rates were rather poor. This may be explained by the lower median Karnofsky Performance State and oncologic status of the majority of patients in our study, so classified the RPA the half of our patients as class III, i.e., gravely ill. In accordance with the current literature, 65% of our patients died due to extra-cranial progression.

In line with the most oncologic studies, the better median OS of our patients was significantly associated with higher KPS. For this reason, KPS is an important part of all prognostic scores (e.g., Johung et al. 2016).

Other than reported, there was no evidence of death events due to Local Control Failure in our patient collective, where the neurologic death cases (6%, *n* = 2/34) resulted from a distant meningeosis. That may be due to a relatively low total number of patients and a relatively high number of patients with a low performance state.

Our results are in line with the reported LC rates (68.6–87%, related to the number of treated targets) and median survival time range (7–13.9 months) for single shot stereotactic radiosurgery by different modalities (LINAC, Gamma Knife or Cyberknife^®^) (Flickinger et al. 1994; Li et al. 2000; Hoffman et al. 2001; Aoyama et al. 2006; Kocher et al. 2011; Murovic et al. 2017; Brown et al. 2016).

Regarding hypofractionated stereotactic radiosurgery using LINAC, our results are in the range of the reported LC rates (56–88%) and at the lower end of the range for median survival time (6–14.8 months), as well as for the median follow-up (6–17.4 months), due to a preponderance of RPA class III patients in our cohort (Salles et al. 1993; Manning et al. 2000; Feuvret et al. 2014; Rajakesari et al. 2014; Rades et al. 2017; Baliga et al. 2017; Aoyama et al. 2003; Ernst-Stecken et al. 2006; Aoki et al. 2006; Narayana et al. 2007; Kwon et al. 2009; Saitoh aet al/ 2010; Kim et al. 2011; Minniti et al. 2014).

Compared to postoperative fractionated local 3D-conformal radiotherapy (3DRT) for resected BM with a reported range of LC rates of 81.2–91.4% at 12 months, our LC rates are at the lower end (78.6%). Due to a healthier sample, they showed longer median follow-ups (9.7–19.1 months) and survival rates at 12 months (68–77.7%) (Hashimoto et al. 2011; Shin et al. 2015; Igaki et al. 2017; Ayas et al. 2018).

The additional co-treating of small tumors by CK-hSRS proved no disadvantages in our study, as especially smaller tumors showed the tendency to better response and additional treatment modality was spared. This concept simplifies the management, may spare medical resources, and provide additional time for, e.g., a systemic therapy or just improve the quality of life. Backing the above-mentioned tendency to better response of smaller tumors, Lesueur et al. (2018) estimated a significantly decreased risk of local control failure (from 36 to 17% at 12 months, *p* = 0.007) for the CK-hSRS therapy of radio-resistant BM (melanoma and renal carcinoma) showing < 1 cm in diameter and Mengue et al. (2020) showed the target size of < 2.5 cm to be an independent factor improving LC in CK-hSRS therapy of BM (*p* = 0.01).

Among the tumors with local control failure after CK-hSRS (*n* = 6/57) was no adverse tendency for pre-irradiated ones (33%, *n* = 2/6), for location of the targets or for the total number of the targets per patient, but there was a strong tendency for larger BMs, so were all recurrent targets at least 2 cm in the diameter.

Like reported by Nakamura et al. (2017), we detected the most LC failures at the second half year after the treatment. On the contrary, Murai et al. (2014) and Lesueur et al. (2018) observed distinct more LC failures at the first half year.

Interestingly, 67% (*n* = 4/6) of targets with LC failure in our study originated from breast cancer primary, whereas there is an increasing evidence of not existing radio-resistance against radiosurgery (Lesueur et al. 2018). Further statistical estimation of LC failure was not feasible due to a very low number of events.

Our rates of Adverse Events and Toxicities according to CTCAE with 12% Grade 1–3 were moderate compared to the reported range of 5–25% Grade 1–4 (Table 5). They may correlate with extra-large target size and eloquent location.

In addition to the common limitations, as the retrospective character with the risk of hidden selection bias, a small sample size, a heterogeneity of cancer histology, and the treatment indications, there is a reduced FU rate after CK-hSRS in our study with at least 24 of 34 patients (70.6%), due to the poor general condition of our patients and a progressive systemic cancer.

## Conclusion

Outcome reports of CK-hSRS and the reported patient collectives are scarce and heterogeneous to date. This study demonstrated hypofractionated stereotactic radiosurgery using Cyberknife® as an important salvage treatment for cerebral metastases even in pre-irradiated cases. Different Cyberknife^®^ hypofractination schedules proved to be reasonably tolerated and effective. CK-hSRS is a relatively young treatment modality, but it is capable to fill the gap between surgery, single-fraction stereotactic radiosurgery, fractionated radiotherapy, and pure palliative care.

## Data Availability

Datasets generated for this study are available upon request to the corresponding author.
